# Hampden Sidney College, at Richmond, Va.

**Published:** 1848-11

**Authors:** 


HAMPDEN SIDNEY COLLEGE, AT RICHMOND, VA.

We have learned with regret that Dr. Cullen, Professor of the Theory and Practice of Medicine in Hampden Sidney College, has been obliged to relinquish the duties of the chair for the present, on account of indisposition. The institution has been fortunate, however, in obtaining the services of Dr. Meredith Clymer, of this city, for the present session. Dr. Clymer is an accomplished and well-read physician, was formerly editor of this journal, more recently Professor of the Practice of Medicine in the Franklin Medical College of Philadelphia, and is advantageously known to the profession by his valuable contributions to medical literature.

The Faculty of Hampden Sidney have been amongst the most strenuous defenders of the doctrine that students should study only in a southern school, to be qualified to treat southern diseases, but we are glad to find that the disqualification only extends to *schools*, and does not include *professors*. Our acquaintance with Professor Clymer enables us to say, that a short time will suffice to prove to his colleagues that he is entirely capable of treating, and teaching others how to treat, the diseases of that or any other section of the country.

